# Single-cell Mayo Map (scMayoMap): an easy-to-use tool for cell type annotation in single-cell RNA-sequencing data analysis

**DOI:** 10.1186/s12915-023-01728-6

**Published:** 2023-10-20

**Authors:** Lu Yang, Yan Er Ng, Haipeng Sun, Ying Li, Lucas C. S. Chini, Nathan K. LeBrasseur, Jun Chen, Xu Zhang

**Affiliations:** 1 Division of Computational Biology, Department of Quantitative Health Sciences, Mayo Clinic, Rochester, MN 55905 USA; 2 Center for Individualized Medicine, Mayo Clinic, Rochester, MN 55905 USA; 3 Robert and Arlene Kogod Center On Aging, Mayo Clinic, Rochester, MN 55905 USA; 4 Department of Biochemistry and Microbiology, Rutgers University, New Brunswick, NJ 08901 USA; 5 Department of Quantitative Health Sciences, Mayo Clinic, Jacksonville, FL 32224 USA; 6 Department of Physical Medicine and Rehabilitation, Mayo Clinic, Rochester, MN 55905 USA; 7 Department of Biochemistry and Molecular Biology, Mayo Clinic, Rochester, MN 55905 USA

**Keywords:** Single-cell RNA-sequencing, Cell type annotation, Cell type markers, scMayoMap, scMayoMapDatabase

## Abstract

**Background:**

Single-cell RNA-sequencing (scRNA-seq) has become a widely used tool for both basic and translational biomedical research. In scRNA-seq data analysis, cell type annotation is an essential but challenging step. In the past few years, several annotation tools have been developed. These methods require either labeled training/reference datasets, which are not always available, or a list of predefined cell subset markers, which are subject to biases. Thus, a user-friendly and precise annotation tool is still critically needed.

**Results:**

We curated a comprehensive cell marker database named scMayoMapDatabase and developed a companion R package scMayoMap, an easy-to-use single-cell annotation tool, to provide fast and accurate cell type annotation. The effectiveness of scMayoMap was demonstrated in 48 independent scRNA-seq datasets across different platforms and tissues. Additionally, the scMayoMapDatabase can be integrated with other tools and further improve their performance.

**Conclusions:**

scMayoMap and scMayoMapDatabase will help investigators to define the cell types in their scRNA-seq data in a streamlined and user-friendly way.

**Supplementary Information:**

The online version contains supplementary material available at 10.1186/s12915-023-01728-6.

## Background

Tissues are constructed of diverse cell types that support highly specific functions in multicellular species. The development of single-cell sequencing (scRNA-seq) technologies has enhanced our capability to understand the molecular profiles of these individual cells and enabled us to study the heterogeneous cellular composition of complex tissues in the context of development, aging, health, and disease [1, 2]. In scRNA-seq data analysis, cell type annotation is a critical step and can be done manually with sufficient knowledge but is labor intensive and time-consuming. To achieve automatic cell annotation, computational tools have been developed to annotate either cells or cell clusters. Cell-annotation methods such as SingleCellNet [3], SingleR, scmap [4], and Azimuth [5] assign cell identity to individual cells based on a pre-annotated scRNA-seq dataset as a reference or training dataset. However, accurate annotated reference data is not always available. Other tools, such as SCINA [6] and CellAssign [7], assign cell types based on known marker genes but they could be prone to the biases of the markers used. Besides, some deep learning-based, such as scDHA [8] and scBalance [9] are also available, but also require reference data.

In some cases, an annotation tool is accompanied by a cell marker database. For example, scCATCH [10] built a reference database “CellMatch” combining databases CellMarker [11], Mouse Cell Atlas (MCA) [12], CancerSEA [13], and the CD Marker Handbook [14]. SCSA [15] integrated databases from CellMarker and CancerSEA. Some of these databases (e.g., MCA) were derived from differential expression analysis of scRNA-seq data. Other expert-curated databases (e.g., PanglaoDB) were manually curated from thousands of published studies. The annotation accuracy strongly depends on the informativeness and comprehensiveness of the marker gene database. To date, existing databases do not have extensive coverage in tissue types and cell types with good specificity. These obstacles can be challenging for investigators who are new to the scRNA-seq field or have limited background knowledge on the tissue and cell types involved.

To achieve a better cell type annotation outcome, we integrated and refined the available scRNA-seq annotation databases, originating a new database named scMayoMapDatabase*.* In parallel, we developed a new annotation tool, scMayoMap. Together with its own database, scMayoMap provides easy, fast, and accurate cell type annotation without the need to provide cell type markers or pre-annotated single-cell reference datasets. We envision scMayoMap will be of considerable utility for the scientific community.

## Methods

### Construction of scMayoMapDatabase

The data used in scMayoMapDatabase (including tissue, cell type, and marker genes, Table S1) was retrieved from literature and public databases including PanglaoDB [16], Azimuth [5], CellMarker [11], CellMatch [10], and MCA [12]. Marker genes from undefined tissues and cancer cells were not included. Individual tissues and cell types of each organ system were manually reviewed by experts in each organ system. The name of each tissue and cell type from different sources were standardized, and the corresponding cell markers were integrated. Both mouse and human genes were included in the database. Unofficial gene names were revised based on Mouse Genome Informatics [17] and GeneCards [18].

### Cluster annotation process

**Fig. 1:**
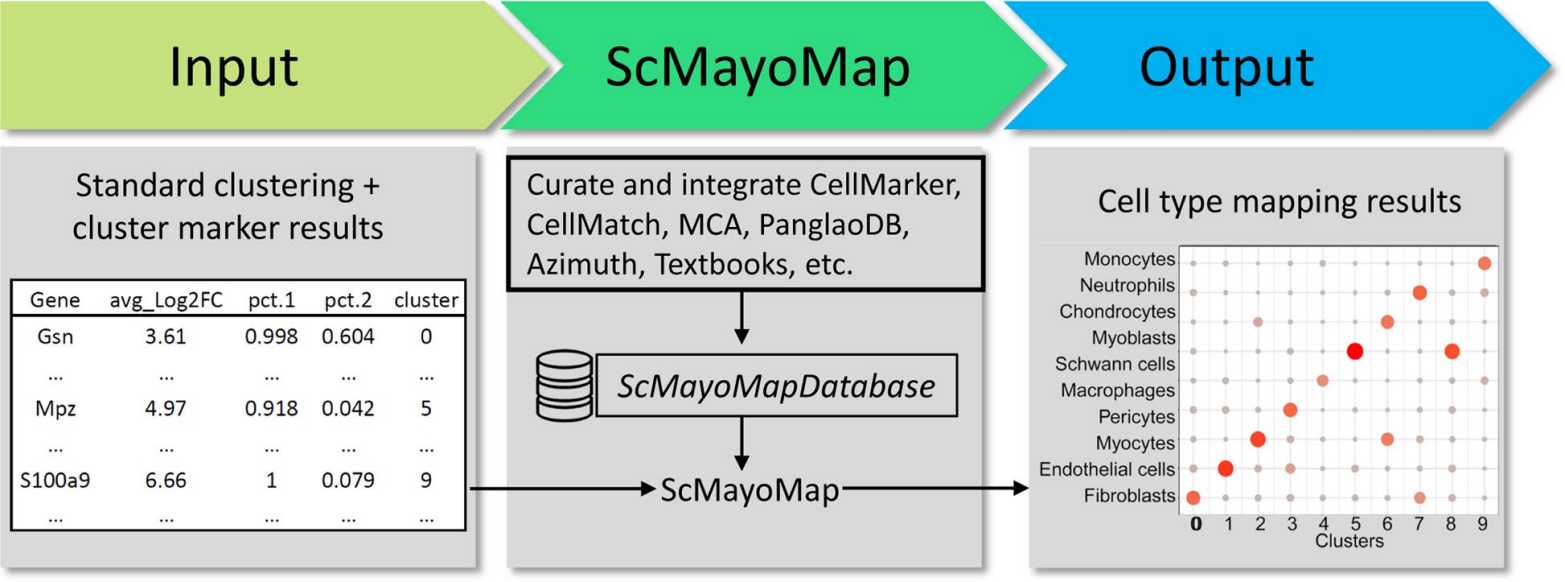
scMayoMap workflow. scMayoMap is at the downstream of a pre-processing, clustering, cluster marker identification. It takes the cluster marker gene list as input and returns the cell type prediction results in a plot and the mapped gene list

The central algorithm for cell type annotation is based on the hypothesis that a good marker gene will be highly expressed in the cell type but not in unrelated cells. Based on this hypothesis, we designed the following steps to annotate cell clusters (Fig. 1).

#### Identification of potential marker genes for each cluster

Cells were first pre-processed using the standard pipeline and clustered using a clustering method, such as Seurat’s default shared nearest neighbor (SNN) modularity optimization-based clustering algorithm. Differential expression analysis is performed to find the marker genes for all $\mathrm{K}$ clusters$.$ Gene expression in each cluster $\mathrm{k }\left(1\le \mathrm{k}\le \mathrm{K}\right)$ is compared to the combination of all other clusters. This process can be easily done by applying the FindAllMarkers function in the Seurat package [5].

#### Cluster annotation with potential marker genes

scMayoMap uses marker genes with percentage of cells expressing the marker gene greater than $0.25$ and adjusted p-value less than $0.05$. For cluster $k$, marker gene $i$, we produce a composite expression score ${S}_{i}^{k}$ synthesizing both the fold change and the prevalence change $:{S}_{i}^{k}=\frac{{2}^{{l}_{i}^{k}}\times {p}_{i}^{k}}{{p}_{i}^{{K}^{\neg k}}}\left(1\le i\le {I}_{k}, 1\le k\le K\right)$, where ${l}_{i}^{k}$ denotes the average log_2_ fold change of marker gene $i$ in cluster $k$ compared to other clusters. ${p}_{i}^{k}$ denotes the percentage of cells in cluster $k$ where the gene $i$ is detected, and ${p}_{i}^{{K}^{\neg k}}$ denotes the percentage of cells in all other clusters where the gene $i$ is detected. The logarithm of ${S}_{i}^{k}$ is essentially the sum of the log fold change and log prevalence change. After that, scMayoMap matches the marker genes in cluster $k$ to tissue-specific cell markers from scMayoMapDatabase and produces a score for each potential cell type $c (1\le c\le C)$ and cluster$k (1\le k\le K)$, which is determined as follows:

$${S}_{c}^{k}=\frac{1}{\begin{array}{c}{I}_{k}\\ \end{array}}\sum\nolimits_{i=1}^{{I}_{k}}{S}_{i}^{k}\times {d}_{ic} \left(1\le i\le {I}_{k}, 1\le k\le K, 1\le c\le C\right),$$

where ${d}_{ic}$ is a binary indicator indicating the absence (${d}_{ic}$ = 0) and presence (${d}_{ic}$ =1) of the marker gene $i$ in the cell type $c$.

To be more robust, scMayoMap allows assignment of multiple cell types to the same cluster if their evidence is similar. Specifically, the predicted cell types for cluster $k$ are determined by the top $n \left(1\le n\le C\right)$ of ${S}_{c}^{k}$, where $n$ is determined by finding the jump point in the cumulative variance of ${S}_{c}^{k}$ (see details in Additional File 1).

#### Visualization of the prediction results

scMayoMap.plot function takes the cell-type prediction scores for each cluster as the input and produces a dot plot showing the predicted cell types for each cluster.

### Experimental datasets

We retrieved 48 scRNA-seq datasets across different platforms and tissues from public resources (see details in Table S2). These datasets were generated by six different platforms, including Smart-seq2, CEL-Seq2, 10 × Chromium, Drop-Seq, Seq-Well, and inDrops protocols [19]. Eighteen tissue types were included for the analysis, including blood, bladder, brain, adipose tissue, heart, kidney, gastrointestinal tract, muscle, liver, lung, mammary gland, bone marrow, pancreas, skin, spleen, thymus, trachea, and tongue [20, 21]. The cell types annotated in the source datasets were used as the benchmarks.

### Comparison between different annotation tools

Four scRNA-seq-specific annotation tools, including two cell annotation methods (scmap [4] and singleR [22]) and two cluster annotation tools (scCATCH [10] and SCSA [15]) were compared. All methods were run with their default settings or based on the provided vignette describing the procedures. Briefly, for scmap, we used the scmap-cluster projection strategy that map the experimental dataset to “pbmcsca” reference dataset from SeuratData package. For SingleR, the HumanPrimaryCellAtlasData was used as the reference, and “label.main” was specified as the prediction level. For scCATCH, tissue and species were specified for the analysis. For SCSA, the default database, whole.db, was used with input file generated by FindMarkers function of Seurat. For ScType, the default database, ScTypeDB was applied. SCINA does not have its own database, where ScTypeDB was applied as the default marker list. To keep method evaluation on the same level, we used the proportion of cells that were correctly annotated as a metric. For cluster annotation methods, the predicted cell type with the maximum prediction score was chosen as the final predicted cell type. The annotation accuracy is the percentage of cells with correct cell type labels based on the source datasets. Cell types in source datasets with ambiguous cell type names, such as pp, MHC class II, PSC, co-expression, immune other and unclassified, were excluded from evaluation.

## Results

### Construction of scMayoMapDatabase

**Fig. 2:**
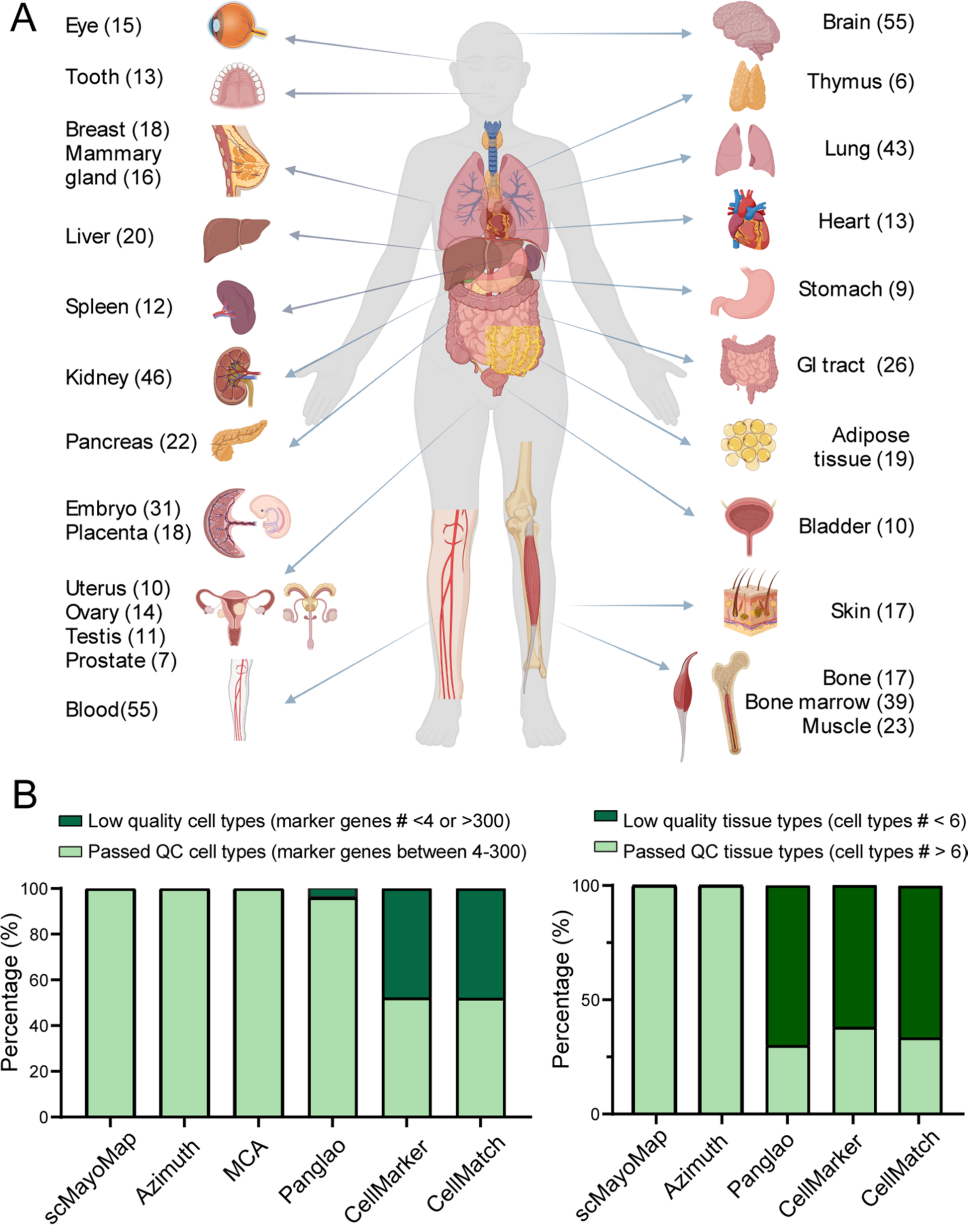
Summary of the scMayoMapDatabase. **A** The scMayoMapDatabase contains 28 tissues and 340 cell types for human and mouse. The number of cell types within each tissue was labeled in parentheses. **B** Comparison of scMayoMapDatabase to other public databases. The left panel shows the cell type information and the right panel shows the tissue information in each database

scMayoMapDatabase covers 340 cell types from 28 tissues and contains a total of 26,487 cell markers for both human and mouse (Fig. 2A and Table S1). It contains data for 12 organ systems including the integumentary system (skin), musculoskeletal system (bone, muscle), nervous system (brain), cardiovascular system (heart), circulatory system (blood), respiratory system (lung), digestive system (tooth, esophagus, liver, pancreas, stomach, small intestine, large intestine), urinary system (bladder, kidney), reproductive system (breast, ovary, placenta, uterus, mammary gland, embryo, testis, prostate), lymphatic system (bone marrow, thymus, spleen), endocrine system (adipose tissue), and visual system (eyes). The annotation level of the collected cell types for most tissues remains at the first level of the hierarchy. Cell markers for each cell type range from a minimum of 4 up to 300, with a mean of 43.8. The quality of the existing databases is partly compromised by tissues with very few cell types and cell types with very few marker genes. These limitations increase the noise for cell type annotation. Most collections in scMayoMapDatabase passed the quality control with more than 6 cell types per tissue and more than 4 marker genes per cell type. The Azimuth database has a good data quality, but the coverage of tissue types is relatively low (10 tissue types) (Fig. 2B).

### scMayoMap excelled in evaluation across multiple tissues and platforms

**Fig. 3:**
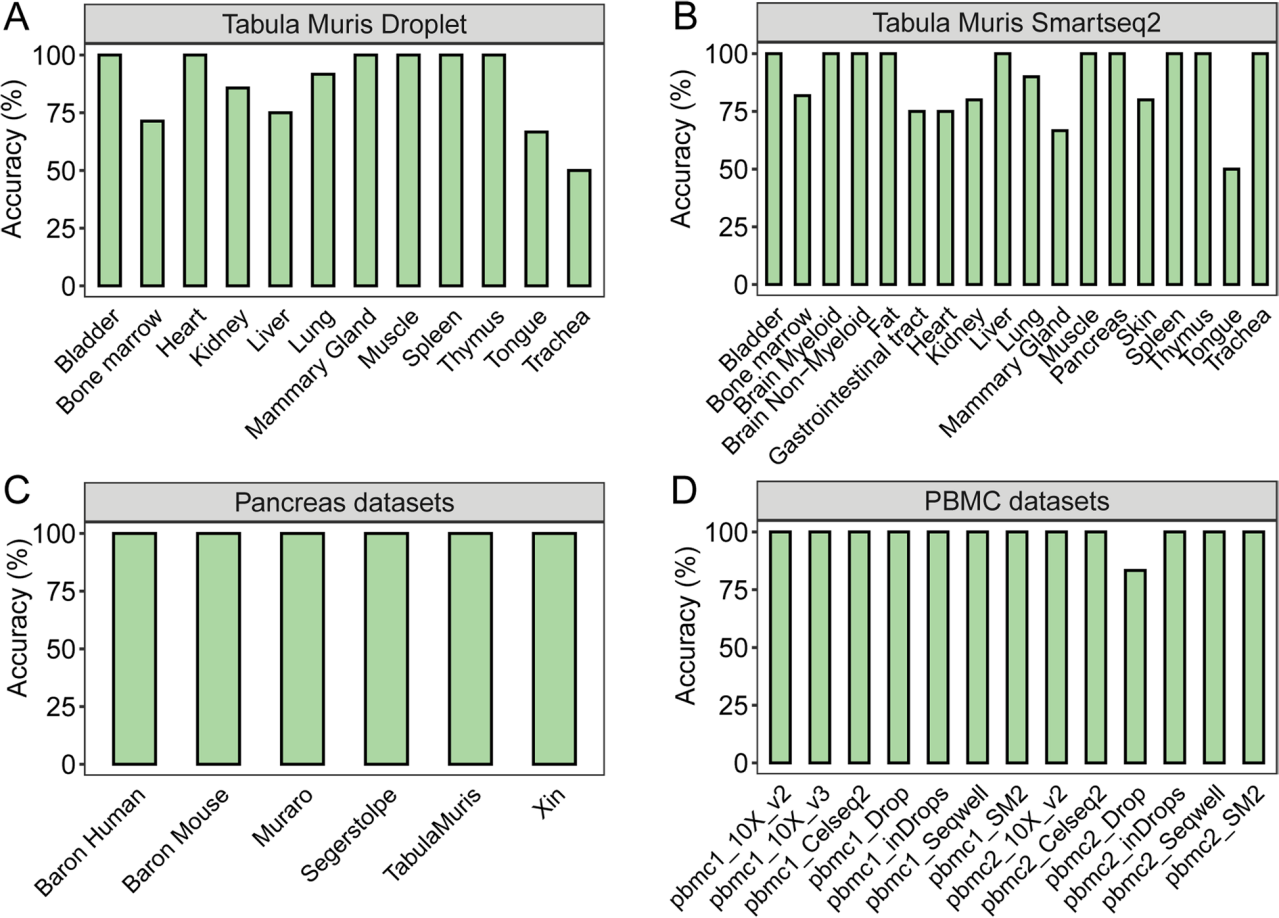
Performance of scMayoMap on experimental datasets cover different tissues. Barplot shows the annotation accuracy of scMayoMap across **A** 12 different tissues on the Tabula Muris Droplet scRNA-seq datasets, **B** 18 tissue types on the Tabula Muris Smartseq2 scRNA-seq datasets, **C** 6 different pancreas scRNA-seq datasets from literature, and **D** 13 different PBMC scRNA-seq datasets. Accuracy is calculated as percentage of clusters correctly annotated by each method

To evaluate the annotation accuracy of scMayoMap on a broad range of tissues, we retrieved data from the Tabula Muris [23] and literature [20, 24–28], covering 48 different scRNA-seq datasets, 18 different tissues, and 6 different scRNA-seq platforms (Table S2). scMayoMap annotated cell types from different tissues with high accuracy (Fig. 3). Specifically, it correctly annotated all clusters in 33 of 48 scRNA-seq datasets. Tabula Muris contains two different datasets that either use the droplet method or the Smartseq2 method. For droplet-based datasets, scMayoMap successfully identified all cell types within tissues including bladder, heart, muscle, mammary gland, thymus, and spleen (Fig. 3A). For Smartseq2-based datasets, more tissues were annotated accurately including bladder, brain, fat, limb, liver, pancreas, spleen, thymus, and trachea (Fig. 3B). When we compared the different pancreas datasets, scMayoMap successfully annotated all datasets with 100% accuracy. scMayoMap correctly identified a, b, d, e, and g cells of the islet, as well as acinar cells, pancreatic progenitor cells, ductal cells, endothelial cells, macrophages, mast cells, stellate cells, Schwann cells, B cells, and T cells (Fig. 3C).

A great amount of scRNA-seq data has been generated in peripheral blood mononuclear cells (PBMCs) because of the high interest and accessibility in both basic and clinical research. Next, we performed a second level of annotation to identify the subpopulations of immune cells in PBMCs, such as CD4 naïve T cells, CD4 memory T cells, CD8 naïve T cells, CD8 central memory T cells, CD8 effector memory T cells, etc. Within 12 of the 13 tested PBMC datasets, scMayoMap correctly identified all cell types with accurate annotation of subtypes in T cells and monocytes. Only one cluster was mis-annotated by scMayoMap in the pbmc2_Drop dataset (Fig. 3D). A good annotation tool should be compatible with different scRNA-seq protocols. These 13 PBMC datasets were generated by 7 types of sequencing technologies, including two low-throughput plate-based methods (Smart-seq2 and CEL-Seq2) and five high-throughput methods (10 × Chromium, Drop-seq, Seq-Well, and inDrops). scMayoMap performed well across all these different scRNA-seq platforms, indicating that it is highly accurate and applicable to a wide range of tissues using different scRNA-seq methods.

### The performance of scMayoMap is superior to other annotation tools

**Fig. 4:**
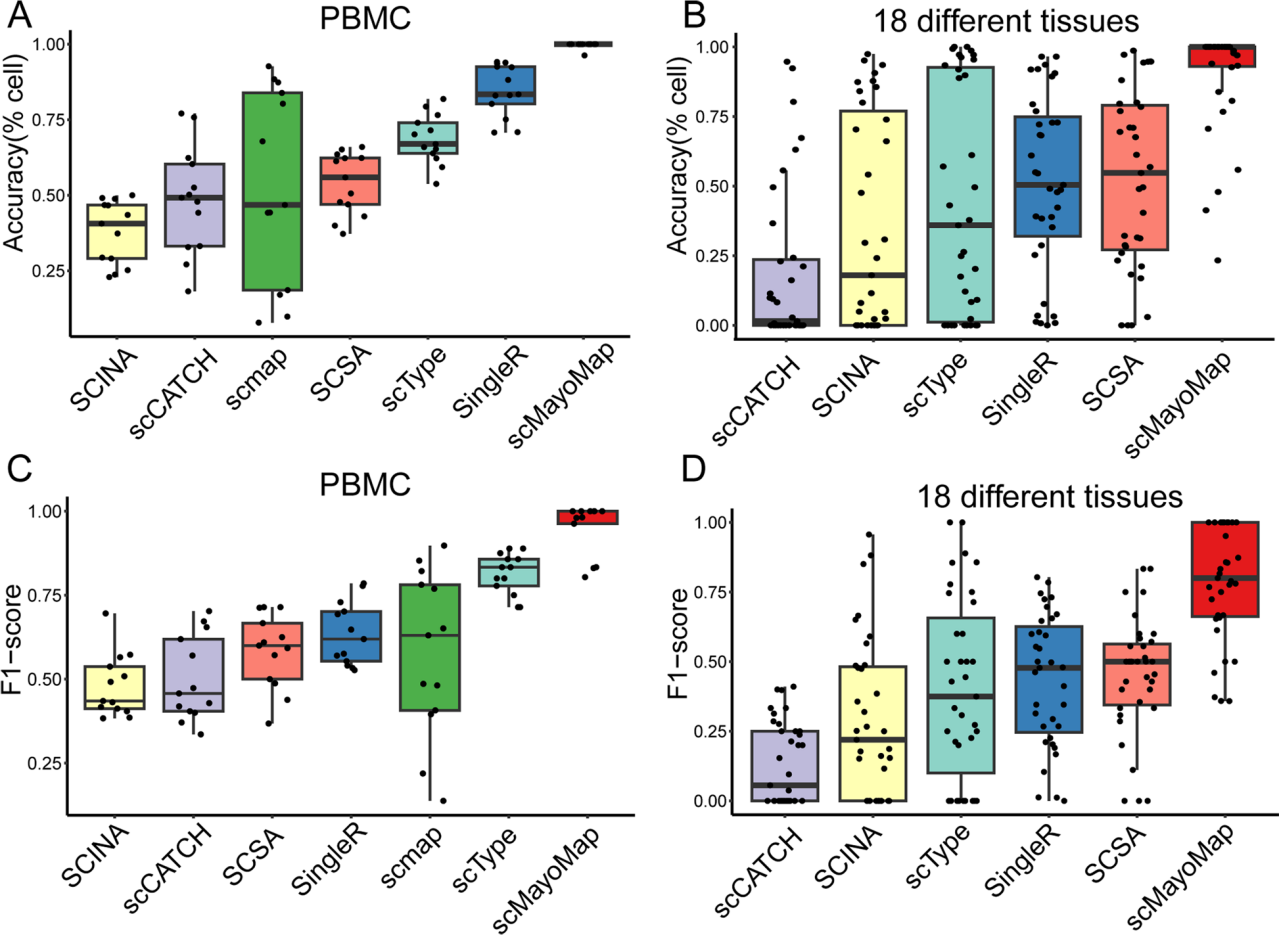
Comparison of scMayoMap and other cell annotation tools on 48 real datasets. **A** Comparison of cell type annotation accuracy in PBMC datasets. Thirteen PBMC scRNA-seq datasets generated by seven sequencing technologies were tested, including two low-throughput plate-based methods (Smart-seq2 and CEL-Seq2) and five high-throughput methods (10 × Chromium, Drop-seq, Seq-Well, and inDrops). **B** Comparison of cell type annotation accuracy on 35 datasets with different tissues from Tabula Muris and literature. The accuracy of each method is presented as the percentage of cells that were correctly annotated by each method. **C** and **D** F1-score calculated for data in **A** and **B**. Jitters on the plot represent datasets. The whisker extends from the hinge to the value that is within 1.5 * interquartile range (IQR) of the hinge, where IQR is the inter-quartile range or distance between the first and third quartiles

To compare the performance of scMayoMap with other available annotation tools, we firstly compared the performance of scMayoMap to SingleR, SCSA, scCATCH, ScType, SCINA, and scmap on the 13 PBMC datasets. As a result, scMayoMap successfully identified all cell types in most of the datasets with a mean accuracy of 99% (range: 83–100%, median: 100%), comparing to 85% for SingleR, 69% for ScType, 54% for SCSA, 53% for scmap, 49% for scCATCH, 38% for SCINA (Fig. 4A).

Further, we assessed the annotation accuracy of scMayoMap and SingleR, SCSA, ScType, SCINA, and scCATCH on 35 datasets from 18 tissues of Tabula Muris datasets and five additional datasets from literature (Fig. 4B). The findings indicated that scMayoMap outperforms other methods, with a mean accuracy of 90% (50–100%, median: 100%). In contrast, SCSA showed a mean accuracy of 52% (0–99%, median: 52%), SingleR showed a mean accuracy of 51% (0–97%, median: 50%), scCATCH showed a mean accuracy of 19% (0–95%, median: 2%), ScType showed a mean accuracy of 44% (0–100%, median: 36%) and SCINA showed a mean accuracy of 36% (0–97%, median: 18%). When evaluated using the F1-score metric, scMayoMap continued to surpass other methods in performance (Fig. 4C and D).

Moreover, scMayoMap was used in multiple different tissues, including mouse skeletal muscle [29], brain [30], and kidney [31], in datasets generated in our lab and provided perfect cell annotation accuracy. Overall, the accuracy of scMayoMapDatabase and scMayoMap is superior to the existing databases and tools.

**Fig. 5:**
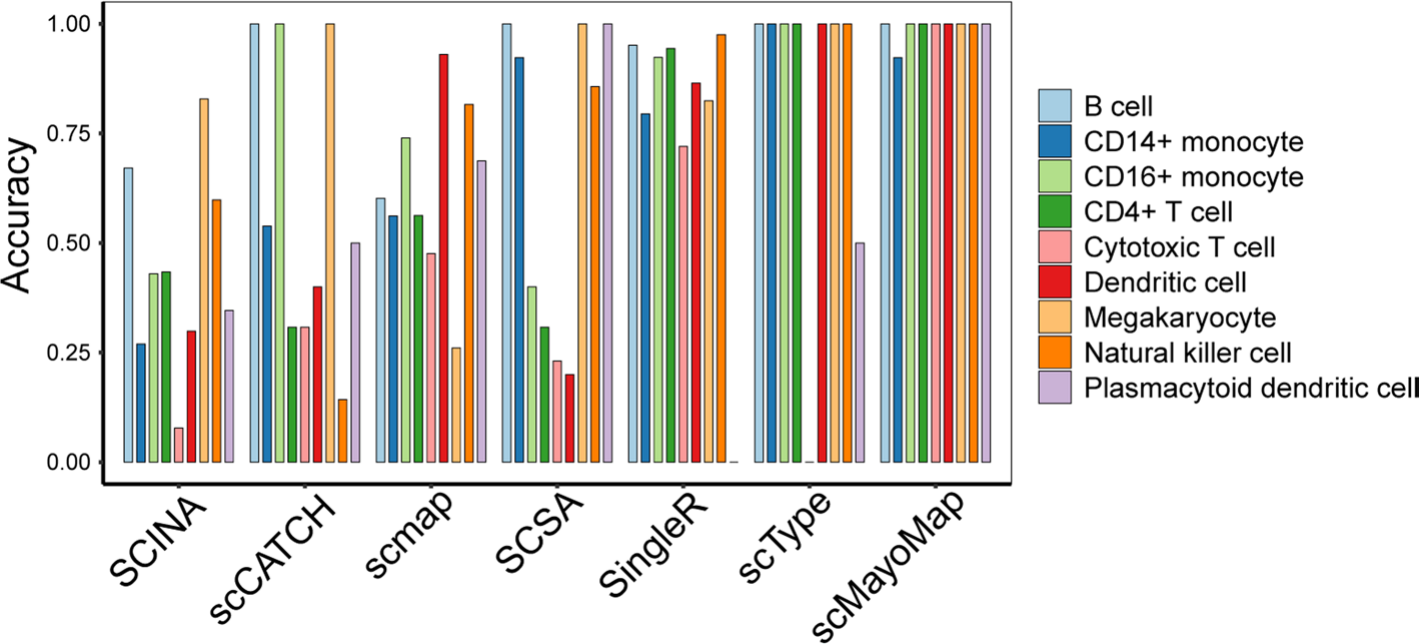
Annotation accuracy for different cell types. Results are evaluated on 13 PBMC datasets. For SCINA, scmap, and SingleR, accuracy refers to the average proportion of cells accurately labeled across 13 datasets. For the other methods, accuracy indicates the percentage of datasets in which cell types were correctly identified

To conduct a more comprehensive evaluation of annotation accuracy across various cell types, we employed the PBMC datasets as illustrated in Fig. 4A. Subsequently, accuracy measurements were computed for each distinct cell type. The outcomes, as depicted in Fig. 5, unmistakably indicate that scMayoMap outperforms other tools in terms of annotation quality across the majority of cell types.

**Fig. 6:**
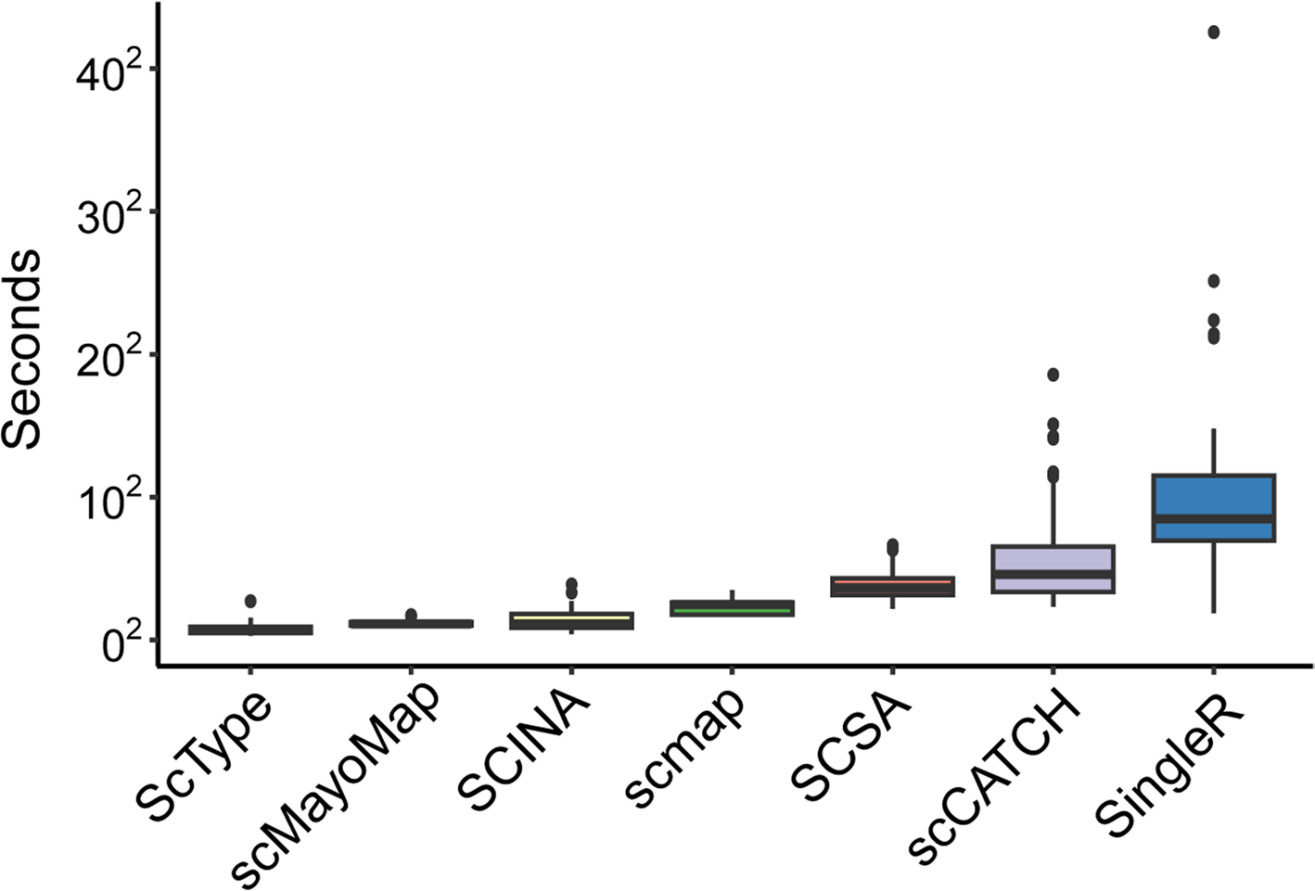
Comparison of computational time between scMayoMap and its competing methods across all datasets evaluated in this study. For scmap, the timing evaluation was only performed on the 13 PBMC datasets, while all other methods covered all 48 datasets

In terms of processing efficiency, we note that scMayoMap performs well (Fig. 6). All calculations are completed within one minute on a MacBook Pro laptop (System Version: macOS 13.5.1, Kernel Version: Darwin 22.6.0, CPU: Apple M2 Pro, Memory: 16 GB).

### scMayoMapDatabase can improve the annotation accuracy of other tools

**Fig. 7:**
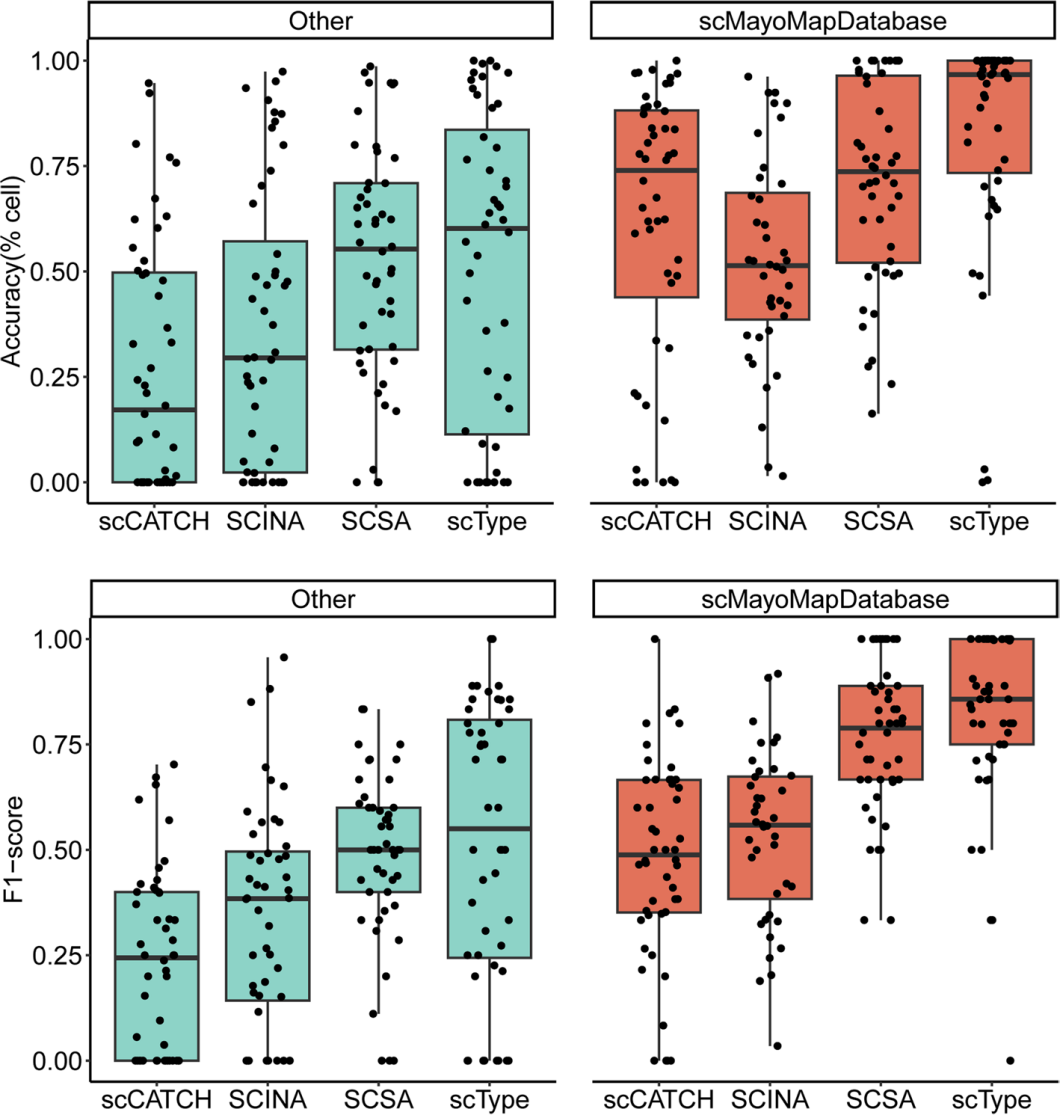
scMayoMapDatabase improves the annotation accuracy (up panel) and F1-score (bottom panel) of its competing methods. The left panels (“Other”) showcase the efficacy of scCATCH, SCSA, and ScType when leveraging their inherent databases, with SCINA utilizing the ScType database. The right panels (“scMayoMapDatabase”) display the performance outcomes of these techniques when they employ the scMayoMapDatabase. These evaluations are based on the 48 datasets assessed in this study

We made scMayoMapDatabase easily accessible from the package and evaluated its performance as a replacement for the internal database of other annotation tools. To test this, we carried out a comparison to discern the efficacy of these methods using their default databases against the scMayoMapDatabase. SCINA doesn't have its own embedded database. As depicted in Fig. 4, ScType outperformed the other methods. Consequently, we integrated SCINA with the internal database of ScType. Interestingly, this led a substantial improvement in the performance of all the all tools; the prediction accuracy and F1-score all increased (Fig. 7). These results suggest that scMayoMapDatabase contributes significantly to the high performance of scMayoMap, and it can be a valuable resource for cell type annotation in scRNA-seq analysis.

## Discussion

We developed a new scRNA-seq data annotation tool, simplifying the cell type annotation process for scRNA-seq data. By using standard clustering results from upstream analysis as input, scMayoMap can swiftly annotate the corresponding cell types for each cluster, based on its internal cell marker database, scMayoMapDatabase. Through testing experimental datasets with varying cell numbers and types, we found that scMayoMap can complete annotation in under a minute by running a single function line included in the scMayoMap package. This enhanced usability of the tool is particularly beneficial for researchers, including those in clinical settings.

The reference database plays a critical role in cell type annotation tools. While several existing cell marker databases have helped advance research using scRNA-seq, they are not without limitations, such as inadequate or inappropriate cell markers and insufficient cell types or tissue coverage. Additionally, there are numerous marker genes in these databases that are identified only by their nicknames, which lack standardization. To overcome these shortfalls, we generated the scMayoMapDatabase by integrating marker genes from current popular databases and manually curating the tissues as well as the cell types. This curated database serves as the foundation for accurate cell type prediction, as demonstrated in our results.

By extensive evaluation on experimental datasets using different scRNA-seq techniques, we demonstrated that the performance of scMayoMap surpassed other methods, including cluster annotation methods and the cell annotation methods. A previous study illustrated that current methods using prior knowledge of marker genes did not show promising performance on PBMC datasets [20]. In this study, we demonstrate that scMayoMap can predict PBMC cell types with small errors, suggesting that a marker-based approach is still a promising approach if applied properly. Additionally, by extensive evaluation on experimental datasets of different tissues, we demonstrate that scMayoMap is a useful tool for cell type annotation with high accuracy.

A typical scRNA-seq data analysis pipeline is several key steps, including pre-processing, clustering, identifying cluster markers, and annotating cell types. We acknowledge that upstream analytical steps, like normalization, clustering, and differential expression analysis, can indeed influence the results of cell type annotation. scMayoMap specifically focus on the annotation step. We acknowledge that scMayoMap has certain limitations, such as its inability to identify new cell types since its predictions rely on an existing marker database. However, it can still provide insights for identifying novel cell types by returning the closest cell types and their corresponding marker genes. We also caution users to be careful when interpreting the prediction results of a cluster that has multiple cell types assigned to it. In order to assist this, we have incorporated an output of evaluation score for clusters with multiple cell types assigned. This score can be easily accessed using the “scMayoMap.obj$markers” command as explained in the package tutorial. Additionally, users can use their biological knowledge to analyze the expression of marker genes retrieved for each cluster to gain further insights. Finally, the precision of scMayoMap is contingent upon the comprehensiveness of the marker gene list generated from differential expression analysis. As such, it is crucial to ensure that the sequencing depth, the count of identified genes, and the cell numbers are not suboptimal, in order to maintain adequate statistical power for identifying marker genes. To ascertain the requisite sequencing depth, gene count, and cell numbers essential for reliable cell type annotation, we recommend that users perform independent power analyses on their specific datasets. This will help in determining the cell count thresholds that are needed for robust cell type annotation.

## Conclusions

Overall, scMayoMap, combined with the comprehensive scMayoMapDatabase, is a user-friendly and powerful tool for annotating cell types in scRNA-seq data analysis, with potential applications in scRNA-seq studies.

### Supplementary Information

**Additional file 1: Table S1.** scMayoMapDatabase created in this study. **Table S2.** Experimental datasets used in evaluation of scMayoMap.

## Data Availability

scMayoMap is an open-source software, available at GitHub (https://github.com/chloelulu/scMayoMap) [32]. The scMayoMapDatabase and raw data for each figure can be found in the Additional File 1: Tables S1-S2. Code analyzed the dataset in this study can be found at (https://github.com/chloelulu/scMayoMap-paper) [33]. Experimental datasets used in this study have been deposited on Figshare [34].

## References

[1] Guo X (2018). Global characterization of T cells in non-small-cell lung cancer by single-cell sequencing. Nat Med.

[2] Kim C (2018). Chemoresistance evolution in triple-negative breast cancer delineated by single-cell sequencing. Cell.

[3] Tan Y, Cahan P (2019). SingleCellNet: a computational tool to classify single cell RNA-seq data across platforms and across species. Cell Syst.

[4] Kiselev VY, Yiu A, Hemberg M (2018). scmap: projection of single-cell RNA-seq data across data sets. Nat Methods.

[5] Hao Y (2021). Integrated analysis of multimodal single-cell data. Cell.

[6] Zhang Z (2019). SCINA: a semi-supervised subtyping algorithm of single cells and bulk samples. Genes (Basel).

[7] Zhang AW (2019). Probabilistic cell-type assignment of single-cell RNA-seq for tumor microenvironment profiling. Nat Methods.

[8] Tran D (2021). Fast and precise single-cell data analysis using a hierarchical autoencoder. Nat Commun.

[9] Cheng Y (2023). A scalable sparse neural network framework for rare cell type annotation of single-cell transcriptome data. Commun Biol.

[10] Shao X (2020). scCATCH: automatic annotation on cell types of clusters from single-cell RNA sequencing data. iScience.

[11] Zhang X (2019). Cell Marker: a manually curated resource of cell markers in human and mouse. Nucleic Acids Res.

[12] Han X (2018). Mapping the Mouse Cell Atlas by Microwell-Seq. Cell.

[13] Yuan H (2019). CancerSEA: a cancer single-cell state atlas. Nucleic Acids Res.

[14] BD Biosciences, BD CD Marker Handbook. www.bdbiosciences.com.

[15] Cao Y, Wang X, Peng G (2020). SCSA: a cell type annotation tool for single-cell RNA-seq data. Front Genet.

[16] Franzén O, et al. PanglaoDB: a web server for exploration of mouse and human single-cell RNA sequencing data. Database (Oxford). 2019;2019:baz046. 10.1093/database/baz046 PMC6450036 30951143

[17] Bult CJ, et al. Mouse Genome Database (MGD) 2019. Nucleic Acids Res. 2019;47:D801–6. 10.1093/nar/gky1056 PMC6323923 30407599

[18] Safran M, et al. GeneCards Version 3: the human gene integrator. Database (Oxford). 2010;baq020. 10.1093/database/baq020 PMC2938269 20689021

[19] Ding J, et al. Systematic comparison of single-cell and single-nucleus RNA-sequencing methods. Nat Biotechnol. 2020;38:737–46. 10.1038/s41587-020-0465-8 PMC7289686 32341560

[20] Abdelaal T, et al. A comparison of automatic cell identification methods for single-cell RNA sequencing data. Genome Biol. 2019;20:194. 10.1186/s13059-019-1795-z PMC6734286 31500660

[21] Soneson C. TabulaMurisData: 10x and SmartSeq2 data from the tabula muris consortium. R package version 1.17.0., 2022.

[22] Aran D (2019). Reference-based analysis of lung single-cell sequencing reveals a transitional profibrotic macrophage. Nat Immunol.

[23] Tabula Muris C (2018). Single-cell transcriptomics of 20 mouse organs creates a Tabula Muris. Nature.

[24] Baron M (2016). A single-cell transcriptomic map of the human and mouse pancreas reveals inter- and intra-cell population structure. Cell Syst.

[25] Muraro MJ (2016). A single-cell transcriptome atlas of the human pancreas. Cell Syst.

[26] Segerstolpe A (2016). Single-cell transcriptome profiling of human pancreatic islets in health and type 2 diabetes. Cell Metab.

[27] Xin Y (2016). RNA sequencing of single human islet cells reveals type 2 diabetes genes. Cell Metab.

[28] Liu F (2019). Systematic comparative analysis of single-nucleotide variant detection methods from single-cell RNA sequencing data. Genome Biol.

[29] Zhang X (2022). Characterization of cellular senescence in aging skeletal muscle. Nat Aging.

[30] Zhang X (2022). Rejuvenation of the aged brain immune cell landscape in mice through p16-positive senescent cell clearance. Nat Commun.

[31] Li LX (2022). Single-cell and cell chat resolution identifies collecting duct cell subsets and their communications with adjacent cells in PKD kidneys. Cells.

[32] Yang L, et al. Single-cell Mayo Map (scMayoMap): an easy-to-use tool for cell type annotation in single-cell RNA-sequencing data analysis. https://github.com/chloelulu/scMayoMap. 10.1186/s12915-023-01728-6 PMC10588107 37858214

[33] Yang L, et al. Single-cell Mayo Map (scMayoMap): an easy-to-use tool for cell type annotation in single-cell RNA-sequencing data analysis. https://github.com/chloelulu/scMayoMap-paper. 10.1186/s12915-023-01728-6 PMC10588107 37858214

[34] Yang L, et al. Single-cell Mayo Map (scMayoMap): an easy-to-use tool for cell type annotation in single-cell RNA-sequencing data analysis. 10.6084/m9.figshare.24239773.v1. 10.1186/s12915-023-01728-6 PMC10588107 37858214

